# INNOVATING HOME HEALTH DELIVERY TO IMPROVE VETERANS’ ACCESS TO CARE AND CARE COORDINATION

**DOI:** 10.1093/geroni/igad104.3774

**Published:** 2023-12-21

**Authors:** Heather Benzel, Jammie Carter, Dina Censky, Daniel Janssen, Rachel Murphy, Leann Putz, Heather Davila, Irene San Roman

**Affiliations:** VA Midwest Healthcare Network (VISN 23), Grand Island, Nebraska, United States; VA Iowa City VA Health Care System, Iowa City, Iowa, United States; Sioux Falls VA Health Care System, Sioux Falls, South Dakota, United States; VA Black Hills Health Care System, Fort Meade, South Dakota, United States; Iowa City VA Health Care System, Iowa City, Iowa, United States; Central Iowa VA Health Care System, Des Moines, Iowa, United States; Iowa City VA Health Care System, Iowa City, Iowa, United States; Iowa City VA Health Care System, Iowa City, Iowa, United States

## Abstract

Nearly 350,000 Veterans receive home health services through the Veterans Health Administration (VA) annually. Historically, VA has purchased most home health services from contract agencies in the community. VA leaders have expressed growing concerns about Veterans’ access to home health, especially in rural areas. Coordinating care between VA and contract agencies is an ongoing challenge. To address these challenges, the VA Midwest Health Care Network (VISN 23) developed the VA Home Health (VAHH) pilot program. The program launched at the Iowa City VA and is currently operating at 4 sites in Iowa and South Dakota. Through VAHH, local VA Medical Centers hire home health staff directly (nurses, aides, physical and occupational therapists), rather than purchase these services through contract agencies. In this presentation, we describe program development, challenges, and early evaluation findings. In fiscal year (FY) 2022, 200 Veterans received VAHH. So far in FY2023, over 700 Veterans have been served. Pre/post comparisons involving Veterans receiving VAHH show modest reductions in emergency room visits (727 vs. 664 visits) and inpatient admissions (227 vs. 192 admissions). Overall hospital lengths of stay have reduced more dramatically (2132 vs. 1390 total days). VAHH staff note close communication with Veterans’ primary care teams, and Veterans describe being highly satisfied with the program. Interest in VAHH is growing nationally, particularly as access to home health in many regions is limited. Early lessons from the VAHH pilot program could inform VA decisions about home health delivery and assist other sites in improving care for Veterans.

